# Association of urinary sodium excretion with blood pressure and risk factors associated with hypertension among Cameroonian pygmies and bantus: a cross-sectional study

**DOI:** 10.1186/s12872-018-0787-3

**Published:** 2018-03-07

**Authors:** Daniel Lemogoum, William Ngatchou, Claude Bika Lele, Cecile Okalla, Marc Leeman, Jean-Paul Degaute, Philippe van de Borne

**Affiliations:** 1 Douala School of Medicine and Pharmaceutical Sciences, Douala University, Douala, Cameroon; 2 ULB-Erasme Hospital, Free Brussels University, University, 808, Lennik Road, 1070 Brussels, Belgium; 3 Douala Heart Institute, Douala, Cameroon

**Keywords:** Urinary sodium excretion, Hunter-gatherer lifestyle, Blood pressure, Hypertension, Pygmy, Bantu

## Abstract

**Background:**

High salt intake increases blood pressure (BP) and hypertension risk. This study aimed to examine association of urinary sodium excretion with BP and hypertension correlates among Cameroonian pygmies under hunter-gatherer subsistence mode and Bantus, living in urban area under unhealthy behavioral habits.

**Methods:**

In this cross-sectional cluster sampling study, we randomly enrolled rural pygmies living in Lolodorf and urban Bantus living in Douala. The World Health Organization steps questionnaire was used to collect socio-demographic and lifestyle data. Height, weight, BP and single overnight spot urine samples were obtained in all participants. BP was measured in triplicate. Urinary sodium and potassium excretion was determined by flame photometry. Data were recorded and analyzed using SPSS 16.0.

**Results:**

We included 150 Pygmies and 150 Bantus aged 38 ± 12 years and 33 ± 11 years, respectively (*p* <  0.0001). Compare to Bantus, pygmy’s height and weight were respectively: 1.54 ± 0.09 m vs 1.72 ± 0.12 m; and 54.4 ± 9.2 kg vs 77.2 ± 14.8 kg, all *p* <  0.0001. Age-standardized prevalence of hypertension was 3.3% among Pygmies and 28% among Bantus (*p* <  0.0001). Age-adjusted systolic and diastolic BP were lower in Pygmies than in Bantus (107 ± 12 vs 119 ± 17 mmHg and 71 ± 11 vs 78 ± 13 mmHg respectively, all *P* <  0.0001). BP increased with age but to a lesser extent in Pygmies (all *p* <  0.01). Urinary sodium excretion was lower in Pygmies than in Bantus (46.9 ± 32.4 vs 121.5 ± 61.0 mmol/l, p <  0.0001). Systolic and diastolic BP were positively associated with urinary sodium excretion in Bantus (all *p* <  0.05). In the two groups, urinary potassium excretion was similar, and was not related to blood pressure. In the total study group and in Bantus taken separately, urinary sodium excretion was higher in hypertensive than in normotensive subjects. Multivariable logistic regression showed that urinary sodium excretion, Bantu status and age emerged as independent determinants of hypertension in the whole study group (OR (95%CI): 1.012 (1.005–1.018); 11.408 (3.599–36.165); 1.095 (1.057–1.135) respectively, *p* <  0.0001).

**Conclusion:**

Hunter-gatherer pygmies exhibit low level of urinary sodium excretion related to low rate of hypertension and slower BP increase with age. Salt intake was a major driver of hypertension in our study population. Our findings highlight the need of efforts to implement nationwide prevention programs promoting risk factor screening and healthier lifestyles including reduction of dietary salt intake in Cameroonian.

## Background

Hypertension is the leading cause of cardiovascular diseases (CVDs) morbidity and mortality worldwide [1] and it has an increasing prevalence in most of sub-Saharan Africa (SSA) countries [2, 3]. However, in SSA, there is marked regional variation in the prevalence of hypertension with rates between 15 and 70% [3–6]. In Cameroon, as in several African countries, the prevalence of hypertension is higher in urban than rural regions [7, 8]. This is attributed to the effects of globalization and urbanization which stimulate unhealthy behavioral changes [4, 7–10]. Causal factors for high blood pressure (BP) associated with the rural to urban migration are: reduced physical activity, stress, and modified diets with increased consumption of salt [7–11]. High dietary salt increases BP and is a leading cause of hypertension [12–16]. Conversely, an increase in dietary potassium reduces BP and attenuates the hypertensive effect of high dietary salt [13].

Few studies have been published on salt consumption in SSA with most available data suggesting a lower intake of salt than in other continents [13–17]. However, some research support a greater impact of high dietary salt on BP in SSA, as the black African ancestry populations are more salt sensitive [16, 18]. Globally, a lack of high quality studies creates uncertainty about the overall impact of dietary salt on the health of African peoples [16].

In most of African countries, much of the dietary salt comes from commercial sources [19]. Therefore, some rural hunter–gatherer populations such as Pygmies, who primarily eat foods from natural sources with their staple diet including mainly complex carbohydrates, fruits, vegetables and bushmeat, would be expected to have low levels of salt intake since they would not be exposed to salt widely and inexpensively available from commercial sources as is the case for bantus living in urban environment.

Pygmies are nomadic and are the largest group of mobile hunter-gatherers of Africa [20]. Limited data indicate that Pygmy hunter-gatherer populations are relatively free of CVDs [21] and of their risk factors as well [21–23]. This study aimed to examine association of urinary sodium with BP and factors associated with hypertension among Cameroonian pygmies under hunter-gatherer subsistence mode and Bantus, living separately in urban area under modern unhealthy behavioral habits.

## Methods

### Study population characteristics

#### Pygmies ecologic setting

The hunter-gatherer Cameroonian pygmies live in the tropical dense forest of Southern Cameroon, in Kavabousse and Bipindi villages located in the Lolodorf health district. They live in camps of 30 to 70 households. Their traditional lifestyle is to move frequently through forests from one campsite to another, living in temporary built huts. In line with their nomadism and hunter-gatherer subsistence mode, they have high and vigorous occupational physical activity, characterized by good aerobic fitness. Their staple diet includes complex carbohydrates (eg. plantain and cassava); fruits (especially bananas and bush mangos, in varying proportions); wild vegetables and bushmeat. River and well water, used for drinking, is of poor bacteriological quality. They occasionally drink palm wine and a traditional spirit drink (“harki”; 60% alcohol content). They have limited contact with the Bantu population. Nevertheless, whenever possible, they exchange part of their hunting products for salt, palm oil, knives, fire weapons and bullets. They have limited access to healthcare and education systems [21].

#### Bantus ecologic setting

Bantu participants were recruited at the ‘Cité des Palmiers’ health district, the second largest and populous health district in Douala, located in Nord West of Douala City, the economic capital of Cameroon**.** The ‘Cité des Palmiers’ health district comprises 8 health areas with an estimated population of 423,253 inhabitants according to the 2012 national census. The population is diversified and representative of the different ethnic and socio-cultural groups in the country (except the pygmy community), and comprises: students, employees, traders, civil servants, housewives, and low, middle and high-income earners from public and private activity sectors. The Bantu participants have lived in the Douala region for at least 10 years. They have access to modern health care and education. Their lifestyle is characterized by important sedentary, exposed to socio-economic and professional stress. Their diet consists largely of processed food products containing added sodium, fats and sugars, domestic animal products, eggs, milk, offals, stimulants, sweets; as well as heavy alcoholic and sugar-containing beverages intake. They frequently smoke cigarettes with estimated prevalence of tobacco used of 24.5% among adult population [24] and 11.2% in youth population [25].

### Study design and participant’s selection

From November 2013 to April 2014, we used a cross-sectional study design to recruit the pygmy participants from Baka group living in a rural area (Lolodorf) and Bantu dwellers living in an urban area (Douala), aged between 20 and 62 years old. The survey was conducted by the trained health professionals simultaneously in urban and rural areas. The week preceding the beginning of the survey, the study observers visited the selected households in sequential order in both rural and urban settings to inform participants about the goals, importance and benefits of the study and the instructions on how to participate.

The study sample size was calculated using the following Lorentz formula [26]: N = z^2^ x p (1-p)/e^2^, with z as confidence level, p as prevalence of hypertension and e as precision. Using the recent nationwide prevalence of hypertension of 47.5% reported by Dzudie et al. [27], we calculated a sample size of 310 individuals to provide an estimate with 95% confidence interval and 5% precision for the overall estimate of the prevalence of high BP. Thus, the estimated sample to be approached was 310 individuals; yet by the end, a total of 337 participants (171 Bantus in Douala and 166 pygmies in Lolodorf) were encountered. Participants were recruited using a three-stage sampling strategy with “Cité des Palmiers” (Douala) and Lolodorf health districts as the first strata. Neighbourhood was considered as the second strata: 6 and 4 neighbourhoods were selected randomly from the total numbers of 20 and 12 neighbourhoods (population size of 32 neighbourhoods) in the “Cité des Palmiers” and Lolodorf health districts, respectively. The third stage of sampling consisted of randomly selecting 20 households in each of the 10 selected neighbourhoods (12 and 8 in the “Cité des Palmiers” and Lolodorf health districts, respectively) making a total of 200 eligible households to be included in the survey, assuming three adults aged ≥18 years living in each household. The list of households was obtained from the Cameroon National Institute of Statistics. On five working days/week, team of four trained health interviewers, each including one physician, two nurses, and one medical assistant, systematically visited selected households between 05:30 and 09:00 h. The chiefs of the neighbourhoods assisted in designing an itinerary to visit each neighbourhood to locate the selected households. In total, 3.6% of the selected households were not found either because they had left the neighbourhood or the house could not be located. When a randomly selected household was found, all inhabitants of the household met by the survey officers were invited to participate in the study. When the survey officers missed some participants because they had already left the house, they made up to three subsequent attempts to meet them. Participants aged ≥18 years and who signed the informed consent were included to the survey. Exclusion criteria were known impaired renal function, use of diuretics and pregnancy.

### Data collection and tools

#### Demographic and anthropometric measurements

Data collection and all measurements were performed in the participants’ households. During the home visits, survey officers administered a structured adapted World Health Organization (WHO) steps questionnaire to the participants in a secluded place in the household. The questionnaire assessed socio-demographic variables, education level, personal medical history and use of prescription medication, smoking habits, alcohol intake, and leisure-time physical activity. For all participants, ages were recorded and cross-checked with national identity cards and the birth registry provided by local municipal and health authorities. They underwent biometric measurements including BP, heart rate (HR), waist circumference (WC), weight, and height. The weight was measured with electronic medical scales (Seca, Germany). Height was measured with fixed stadiometers (Seca, Germany). The body mass index (BMI) was calculated as weight in kilograms divided by height squared in meters (kg/m^2^). WC was measured using a scaled band.

#### Blood pressure measurement

BP was assessed according to a published standard [28]. All measurements were performed after 15 min rest in the supine position in a quiet room. Using an automated sphygmomanometer (HEM-705 CP, Omron Corporation, Tokyo, Japan)**.** Brachial systolic BP (SBP) and diastolic BP (DBP) and HR were assessed in the supine position on the right arm three times 5 min apart on one session. The average of the three BP measurements was used. Pulse pressure (PP) was calculated as SBP minus DBP and mean arterial pressure (MAP) as DBP plus one-third of PP.

#### Urine collection samples

A single morning overnight urine samples of at least 8 h fasting time was collected in all participants. The day preceding starting urinary collection, participants were carefully informed, instructed and educated by trained health observers on how to collect the overnight fasting morning urine samples. To guarantee rigorous urinary collection, all procedure was supervised and witnessed by two representatives of the research team and the traditional authorities. All samples were self-collected mid-stream urines by each participant using a sterilized container. They were immediately frozen at − 4 °C using iceboxes and transported within 8 h to the laboratory of the Douala General Hospital (Douala, Cameroon) where they were frozen at − 20 °C and analyzed. Urinary sodium and potassium excretion was measured by flame photometry using an automated analyzer (Cobas C311, Roche, Germany). Participants with proteinuria were excluded from the study.

All the examinations were performed between 7:00 am to 12:00 am to avoid variation of BP parameters. Weight, height and BP measurements were performed by the trained health professionals.

#### Definitions of variables

According to the WHO recommendations [29], Bantus were considered physical active if they reported performing: 1) at least 150 min of moderate-intensity aerobic physical activity throughout the week; or 2) at least 75 min of vigorous-intensity aerobic physical activity throughout the week; or 3) an equivalent combination of moderate and vigorous-intensity activity. Participants were considered as literate if they have ever attended first year of school education. Smoking status was considered for current (regular and occasional) smokers while alcohol intake was defined as having at least one alcoholic drink per week. Overweight was defined as a BMI ≥ 25 kg/m^2^, and obesity was BMI > 30 kg/m^2^, and abdominal obesity was defined as WC ≥ 102 cm for men and WC ≥ 88 cm for women [30]. Hypertension was defined as systolic BP ≥ 140 mmHg and/or diastolic BP ≥ 90 mmHg, or a self-report of taking antihypertensive medication [31].

#### Statistical analysis

Data were recorded and analyzed using the SPSS 16.0 software. Data were compared between pygmies and Bantus using Student t-test (for quantitative variables) and Chi square test (for proportions). For comparison of urinary sodium excretion between normotensive and hypertensive participants, we used a non-parametric median comparison test. Partial Pearson’s correlation test was used to assess the association between urinary sodium and potassium excretion and BP. All correlations between BP and electrolytes were adjusted for age, BMI and WC. Linear regression was used to assess the relation between BP and aging and slopes were compared between pygmies and Bantus using an analysis of covariance (ANCOVA). To assess factors independently associated with hypertension, we used a multivariate logistic regression with a backward stepwise procedure. We firstly performed a univariate logistic regression with demographic and cardiovascular risk factors of the study participants. Only variables with a *p* value < 0.2 were included in the multivariate model and presented here. Accuracy of the model was assessed using the Hosmer-Lemeshow goodness-of-fit test. p value of 0.693 confirmed the accuracy of the model. Odds ratio (OR) and 95% confidence interval (CI) were used to quantify the association between hypertension and related variables. The level of significance was established at *p* <  0.05.

#### Ethical considerations

One month preceding the survey, the communities and their leaders were informed by the study investigators about the goals, the importance and the benefits of the study. Participation in the study was voluntary. All the study participants provided written informed before inclusion in the survey. The study protocol was approved by the Douala University Institutional Research on Human Health Ethic Committee (Registry number CEI-UDO/880/11/13).

## Results

About 21 Bantu participants in Douala and 16 pygmy participants in Lolodorf refused to participate to survey. A total of 150 hunter-gatherer rural pygmies and 150 urban Bantus were finally included in the present survey (Table1). The survey participation rates in Douala and lolodorf were 87.7% and 90.4%, respectively.

The pygmies were older and shorter than the Bantus (all *p* <  0.001). Overall, there were more men than women participants, however the sex ratio did not differ significantly between pygmy and Bantu dwellers (*p* > 0.05) (Tables 1 and 2). Educational level, overweight/obesity and abdominal obesity rates, SBP and DBP, MAP and PP were lower in pygmies than in Bantus (all *p* <  0.01). Tobacco use rate in Bantus was 5.4% and was not significantly different from that of pygmies (6.7%). The age-adjusted prevalence of hypertension was markedly lower in pygmy than in Bantu participants: 3.3% vs 28% respectively; *p* <  0.0001 (Table 1). Pygmy hypertensive participants were newly diagnosed for high BP and were not under antihypertensive medication. Only 18% of all Bantu hypertensive patients of the current study were under antihypertensive medication, of whom 95% reported taking regularly calcium antagonists, while 5% of them were under alpha-methyldopa. Neither calcium channel blockers, nor alpha-methyldopa influence urinary sodium excretion when taken chronically. According to WHO recommendations on physical activity, only 23% of Bantu dwellers were physically active. Heavy alcohol consumption rate was high in the both two study groups, however, it magnitude was more pronounced in Bantu than in pygmy participants (66.7% vs 52%, *p* = 0.014). Urinary sodium excretion was more than twice lower in Pygmies than in Bantus (*p* <  0.0001). By contrast, potassium urinary excretion was similar between the two groups (*p* = 0.3). The sodium to potassium excretion ratio was lower in the pygmies than in the Bantus (*p* <  0.0001) (Tables 1 and 2).

**Table 1:** Socio-demographic, biometric and biologic characteristics of the study population

	Total *N* = 300	Bantus *N* = 150	Pygmies *N* = 150	p
Age, years	35 ± 12	33 ± 11	38 ± 12	**0.0001**
Male gender, %	55	57	52.6	0.4
Married status (%)	161 (53.7)	62 (41.3)	99 (66)	**< 0.0001**
Literate^a^(%)	209 (69.7)	150 (100)	59 (39.3)	**< 0.0001**
Tobacco (%)	10 (3.3)	8 (5.4)	10 (6.7)	0.9
Alcohol (%)	178 (59.3)	100 (66.7)	78 (52.0)	**0.014**
Height, m	1.63 ± 0.14	1.72 ± 0.12	1.54 ± 0.09	**< 0.0001**
Weight, kg	65.8 ± 16.8	77.2 ± 14.8	54.4 ± 9.2	**< 0.0001**
BMI, kg/m^2^	24.4 ± 4.8	26.0 ± 5.6	22.9 ± 3.1	**< 0.0001**
Overweight/obesity (%)	106 (35.3)	71 (47.3)	35 (23.3)	**< 0.0001**
WC, cm	77 ± 11	78 ± 14	75 ± 5	**0.002**
AO (%)	14 (4.7)	13 (8.7)	1 (0.7)	**0.003**
SBP, mmHg	113 ± 16	119 ± 17	107 ± 12	**< 0.0001**
DBP, mmHg	74 ± 12	78 ± 13	71 ± 11	**< 0.0001**
MBP, mmHg	87 ± 12	92 ± 12	83 ± 10	**< 0.0001**
PP, mmHg	39 ± 13	41 ± 14	36 ± 11	**0.002**
Hypertension (%)	47 (15)	42 (28)	5 (3.3)	**< 0.0001**
Na+, mmol/L	84.2 ± 61.4	121.5 ± 61.0	46.9 ± 32.4	**< 0.0001**
K+, mmol/L	70.8 ± 45.2	73.5 ± 44.7	68.2 ± 45.8	0.3
Na+/K+ ratio	2.0 ± 2.6	2.6 ± 3.1	1.3 ± 1.9	**< 0.0001**

^a^attempt at least a first year of school education, *BMI* Body mass index, *WC* Waist circumference, *AO* Abdominal obesity, *SBP* Systolic blood pressure, *DBP* Diastolic blood pressure, *MBP* Mean blood pressure, *PP* Pulse pressure, Quantitative data are presented as mean ± SD and qualitative data are presented as count (percentage)

**Table 2:** Socio-demographic, biometric and biologic characteristics of Pygmy and Bantu participants by gender

		Male			Female	
	Bantus *N* = 86	Pygmies *N* = 79	p	Bantus *N* = 64	Pygmies *N* = 71	p
Age, years	33 ± 11	40 ± 13	**0.0004**	32 ± 11	35 ± 12	0.1
Married status (%)	32 (37.2)	58 (73.4)	**< 0.0001**	30 (46.9)	41 (57.7)	0.207
Literate^a^(%)	86 (100)	26 (32.9)	**< 0.0001**	64 (100)	33 (46.5)	**< 0.0001**
Tobacco (%)	5 (5.8)	10 (12.7)	0.127	1 (1.3)	0	–
Alcohol (%)	58 (67.4)	58 (73.4)	0.5	42 (65.6)	20 (28.2)	0.1
Weight, kg	77.5 ± 13.1	57.4 ± 9.7	**< 0.0001**	76.8 ± 17.0	51.0 ± 7.3	**< 0.0001**
Height, m	1.76 ± 0.12	1.58 ± 0.09	**< 0.0001**	168 ± 0.10	149 ± 0.06	**< 0.0001**
BMI, kg/m^2^	25.2 ± 5.0	22.9 ± 3.3	**0.001**	27. 1 ± 6.2	22.8 ± 3.0	**< 0.0001**
Obesity (%)	13 (15.1)	5 (6.3)	**< 0.0001**	37 (57.8)	17 (23.9)	**< 0.0001**
WC, cm	77.3 ± 12.1	75.5 ± 4.2	0.1	80.0 ± 17.0	73.7 ± 5.8	**0.006**
AO (%)	2 (2.3)	0	0.1	11 (17.2)	1 (1.4)	**0.004**
SBP, mmHg	120 ± 15	108 ± 12	**< 0.0001**	119 ± 19	106 ± 12	**< 0.0001**
DBP, mmHg	79 ± 12	71 ± 11	**< 0.0001**	78 ± 14	70 ± 11	**0.0005**
MBP, mmHg	92 ± 11	83 ± 10	**< 0.0001**	91 ± 14	82 ± 10	**< 0.0001**
PP, mmHg	41 ± 14	37 ± 12	**0.045**	41 ± 16	35 ± 9	**0.014**
Hypertension (%)	22 (25.6)	4 (5.1)	**0.000**	20 (31.3)	1 (1.4)	**< 0.0001**
Na+, mmol/L	113.0 ± 58.4	48.1 ± 36.4	**< 0.0001**	132.8 ± 63.1	45.5 ± 27.5	**< 0.0001**
K+, mmol/L	75.2 ± 45.6	68.9 ± 47.1	0.3	71.3 ± 43.8	67.4 ± 44.7	0.6
Na+/K+ ratio	2.3 ± 3.3	1.3 ± 1.9	**0.018**	2.6 ± 3.0	1.3 ± 1.8	**0.005**

^a^attempt at least a first year of school education, *BMI* Body mass index, *WC* Waist circumference, *AO* Abdominal obesity, *SBP* Systolic blood pressure, *DBP* Diastolic blood pressure, *MBP* Mean blood pressure, *PP* Pulse pressure, Quantitative data are presented as mean ± SD and qualitative data are presented as count (percentage)

### Association between blood pressure and aging

**Fig. 1:**
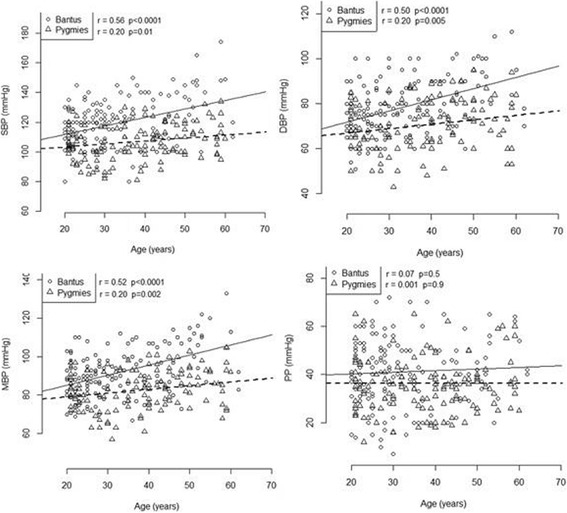
Linear regression of Systolic blood pressures (SBP), Diastolic blood pressure (DBP), Mean blood pressure (MBP) and Pulse pressures (PP) with aging. r represents linear regression coefficient

Apart of PP; SBP and DBP, and MAP significantly increased with aging in both pygmies and Bantus, however, the magnitude of the effects of aging on BP was less prominent in the pygmies than in the Bantus. Indeed, the slopes between SBP, DBP, MAP and age were less steep in the pygmies than in the Bantus (all p <  0.0001) (Fig. 1).

### Comparison of urinary sodium excretion in normotensive and hypertensive study participants

**Fig. 2:**
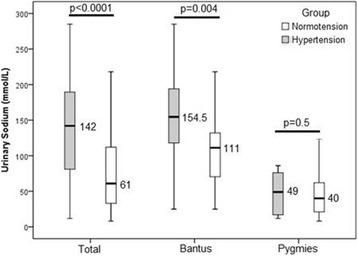
Comparison of urinary sodium between hypertensive and normotensive participants. The midline of the boxes represents the median and the lower and upper margins represent the 25th and 75th percentiles, respectively. The lower and upper ends represent the minimum and maximum values respectively

The mean urinary sodium excretion was higher in hypertensive than in normotensive participants (p <  0.0001). In traditional pygmies group taken separately, urinary sodium excretion did not differ between normotensive subjects and those with hypertension (*p* = 0.5). In the Bantu group, urinary sodium excretion was greater in hypertensive than in normotensive participants (*p* = 0.004) (Fig. 2).

### Relation between blood pressure, and urinary sodium and potassium concentration

There was a significant positive correlation between urinary sodium excretion and SBP, DBP and MAP corrected for age, BMI and WC in the whole study population (all *p* <  0.001). Similarly, urinary sodium excretion was significantly and positively correlated with SBP, DBP, and MBP corrected for age, BMI and WC in the Bantus with a steeper slope (all *p* <  0.05). By contrast, no association was observed between urinary sodium excretion and BP in pygmies (*p* > 0.05). Neither urinary potassium excretion, nor sodium-to-potassium ratio were significantly associated with BP in the whole study group, or in pygmy and Bantu participants taken separately (Table 3).

**Table 3:** Partial correlation coefficients between blood pressure and electrolytes

	SBP, mmHg	DBP, mmHg	MBP, mmHg	PP, mmHg
Total				
Na+, mmol/L	0.266***	0.255***	0.292***	0.080
K+, mmol/L	−0.013	0.049	0.028	−0.060
Na+/K+	0.098	0.020	0.057	0.096
Bantus				
Na+, mmol/L	0.161*	0.213**	0.225**	0.005
K+, mmol/L	−0.032	0.065	0.030	− 0.085
Na+/K+	0.009	− 0.072	− 0.046	0.066
Pygmies				
Na+, mmol/L	−0.143	−0.099	− 0.129	−0.058
K+, mmol/L	−0.008	0.025	0.015	−0.034
Na+/K+	−0.033	−0.067	− 0.062	0.030

*P <  0.05; **p <  0.01; ***p < 0.001

*SBP* Systolic blood pressure, *DBP* Diastolic blood pressure, *MBP* Mean blood pressure, *PP* Pulse pressure

All coefficients are standardized for age, body mass index and waist circumference

### Multivariate logistic regression for the prediction of hypertension

Age (OR 1.095, 95%CI 1.057–1.135, *p* <  0.0001), Bantu status (OR 11.408, 95%CI 3.599–36.165, p <  0.0001), and urinary sodium excretion (OR 1.012, 95%CI; 1.005–1.018, p <  0.0001) emerged as independent determinants of hypertension, while urinary potassium and sodium/potassium ratio were not independently associated with hypertension (Table 4).

**Table 4:** Multivariable logistic regression for the prediction of hypertension in the whole study population

	OR (95% CI)	p
Age, years	1.095 (1.057–1.135)	**< 0.0001**
Bantu status	11.408 (3.599–36.165)	**< 0.0001**
Male gender	0.892 (0.399–1.993)	0.781
Tobacco use: yes	4.813 (0.657–35.231)	0.122
Alcohol intake: yes	0.943 (0.388–2.291)	0.898
BMI, kg/m^2^	0.994 (0.919–1.075)	0.882
WC, cm	1.022 (0.989–1.057)	0.191
Urinary Na+, mmol/L	1.012 (1.005–1.018)	**< 0.0001**
Urinary K+, mmol/L	0.994 (0.982–1.006)	0.301
Na+/K+ ratio	0.917 (0.796–1.057)	0.230

*CI* Confident interval, *BMI* Body mass index, *WC* Waist circumference, *OR* Odds ratio

## Discussion

In this cross-sectional study, we have found marked differences in blood pressure and prevalence of hypertension between Cameroonian pygmies and Bantus. In keeping with the mean high BP and higher prevalence of hypertension in Bantus relative to Pygmies, we found that urinary sodium excretion, alcohol consumption, overweight/obesity, physical inactivity and visceral obesity were also elevated in Bantus, while urinary potassium excretion was similar. Furthermore, we found that sodium excretion was significantly greater in hypertensive than normotensive participants in the Bantus group. This was not the case in pygmies where hypertension was uncommon, urinary sodium much lower and blood pressure increased little with age. Importantly, multivariate logistic regression analysis revealed urinary sodium excretion as an independent determinant of hypertension in the whole study group.

High sodium intakes are related to salt added to commercial foods or that added during food preparation [32, 33]. Pygmies have retained a hunter-gatherer existence. Their diet consists of about 50% meat and 50% plant and fruits and therefore, their main source of sodium is meat [34]. As in our study, Kesteloot et al. [23] has previously demonstrated that sodium excretion was higher in Bantus than pygmies of southern Cameroon. Conversely, urban dwellers consume more salty foods depending on the level of urbanization and the type of population. Indeed, in a study conducted by Tayo et al. [35], among three different black populations, sodium excretion increased across the east-west gradient (e.g., 123 ± 54.6, 134.1 ± 48.8, 176.6 ± 71.0 mmol/L, Nigeria, Jamaica and US, respectively). This east-west gradient is related to the gradient of development between those three countries. This is consistent with the relation between urbanization, nutritional habits and CVDs [11, 35, 36].

We found that sodium excretion was higher in hypertensive than in normotensive participants in the whole study population. When separated into pygmies and Bantus, sodium excretion remained higher in hypertensive than in normotensive Bantus, while the difference was not significant in pygmies. Our observations are consistent with a greater frequency of salt sensitivity previously reported in black hypertensive patients [16, 37, 38]. Furthermore, when adjusted for age, BMI and WC, sodium concentration was significantly and positively correlated with systolic BP, diastolic BP and MBP in the whole study population and in the Bantus group, but not in the Pygmies. These findings are consistent with the known impact of high dietary salt on blood pressure and hypertension [13, 14]. The lack of correlation between blood pressure and sodium excretion could be explained by the low sodium excretion observed in Pygmies. In the international study of electrolyte excretion and blood pressure (INTERSALT), populations with very low sodium consumption (0.2–51.3 mmol/24 h) had a low blood pressure compared to other populations and a slow increase in blood pressure with age [15]. It has been hypothesized that low sodium intakes may reduce the rise in blood pressure upon aging [15, 16].

In our study, we confirm that nearly all the cardiovascular risk factors (hypertension, abdominal obesity, overweight and obesity), as well as their indicative parameters (BP, BMI, WC) were higher in Bantus than in pygmies [21, 23, 28, 39]. Pygmies are nomadic, moving periodically inside the African forest. They have legendary good aerobic fitness, with low body fat and low prevalence of obesity, and low blood cholesterol levels [21, 22, 40]. Moreover, the hunter-gatherer subsistence living of pygmies is hypothesized as one of the key factors involved in their lesser rise in arterial stiffness with aging [21]. Another consequence of the hunter-gatherer lifestyle is a slower increase in blood pressure with aging, as we have observed. A change in the lifestyle can lead to the development of cardiovascular risk factors, as previously observed on Kalahari Bushmen community by Tichelaar et al. [41], as well as in the Kenyan Luo community by Poulter et al. [9]. Otherwise, not all aboriginal communities express a good cardiovascular health. In the indigenous Maori community in New Zealand, CVD is more prevalent in rural Maori in comparison to urban Maori and to non-Maories [42]. These opposite results emphasize the impact of genetic characteristics in the involvement of CVDs [42] and may play an important role in the populations which we investigated. This will require further studies.

Globally, the observed low rate of cardiovascular risk factors among pygmies, contrasting with their high burden in Bantus, suggests the influence of living environments rather than genetic determinants in the development of the increasing burden of hypertension and CVDs in Cameroon. A major factor underlying the increase in CVDs in Bantus, and globally in other Cameroonian populations is rapid and uncontrolled urbanization, which is a driving force for most of the modifiable risk factors such as obesity, hypertension, reduced physical activity, high salt diet, sarcopenia, and increased tobacco and alcohol consumption [4, 7–10, 43, 44]. This survey findings outline the essence of our hunter-gatherer lifestyle and suggest the need to re-align our modern milieu with our traditional lifestyle to improve cardiovascular health. This includes intense lifestyle education, with emphasis on reduced dietary salt intake, more balanced and diversified diets and regular cardiovascular risk factors screening. Our findings also increase knowledge of the health status of pygmies in a country lacking health data and provide strong arguments to policy-decision maker to implement strategies and solutions that combine ecosystem management and adequate health-sector interventions to improve human health and well-being while maintaining a healthy ecosystem.

## Limitations

There are some limitations to our study. First, because cultural, traditional and economic constraints, it was not considered feasible to collect 24 h urine samples to assess sodium and potassium excretion. Other rather recommend instead using a series of 24 h-urine sodium samples since, in general, 6–10 samples of 24 h urine are required to accurately estimate usual salt intake in an individual. Casual urine samples to assess usual dietary sodium is a frail method given most dietary sodium is excreted within 6 h of ingestion and that in many populations; sodium intake varies markedly between meals and days. In Pygmies, diets are relatively constant without large variation in salt contents of foods. Hence a casual urine sample for sodium might be more accurate in Pygmies than in Bantus with highly varied food intake. In mitigation, averaging sodium excretion based on a casual morning overnight urinary sodium excretion samples from a representative large sample of the diverse worldwide population, appeared a reasonable reflection of the population’s average dietary sodium. The second limitation of the present study is the lack of assessing and comparing the CVDs risk factors in hunter-gatherer rural Bantus and hunter-gatherer rural pygmies. This may have helped to better understand CVDs risk factors beyond environmental, behavioral and dietary risk factors such as genetics differences between Bantu and pygmies. Moreover, our survey sample size was small and may not be representative of all Cameroonian pygmy and Bantu communities living in both rural and urban regions of Cameroon. Thus, we cannot rule out the possibility that participants in the study, as well the association with urinary sodium with BP and hypertension, could differ from a broader and more heterogeneous Cameroonian population. Therefore, caution should be exercised when extrapolating our estimates to a broader population in the same and other settings in Cameroon. The third limitation of the survey is that we could not assess the level of physical activity in pygmies using the same criteria as in Bantus. Thus, it could have not been possible to determine the real impact of physical activity on blood pressure and hypertension risk in the whole study population. In mitigation however, we used a multilevel cluster sampling methodology which we believe is an important methodological strength of our study. Importantly, we have further used standardized measurement procedures to collect data in an accurate and reproducible way and used robust analytic methods to generate estimates that will facilitate comparisons with evidence from elsewhere.

## Conclusion

The present study demonstrates that hunter-gatherer living is associated with low level of urinary sodium excretion, which is associated with a low rate of hypertension and a slower increase of blood pressure with age in traditional pygmies. Importantly, high salt diet was an independent driver of high blood pressure in our study population. Our findings underscore the need of public health efforts to implement cost-effective community-based prevention programs to promote risk factors screening campaigns and healthier lifestyles including strategies for reduction of dietary sodium intake to curb the development of hypertension and other non-communicable diseases in Cameroonian.

## Abbreviations

ANCOVA: Analysis of covariance
BMI: Body mass index
BP: Blood pressure
CI: Confidence interval
CVDs: Cardiovascular diseases
DBP: Diastolic blood pressure
HR: Heart rate
MAP: Mean arterial blood pressure
OR: Odds ratio
PP: Pulse pressure
SBP: Systolic blood pressure
SSA: Sub-Saharan Africa
WC: Waist circumference
WHO: World Health Organization
